# Collagenase-Expressing *Salmonella* Targets Major Collagens in Pancreatic Cancer Leading to Reductions in Immunosuppressive Subsets and Tumor Growth

**DOI:** 10.3390/cancers13143565

**Published:** 2021-07-16

**Authors:** Nancy D. Ebelt, Vic Zamloot, Edith Zuniga, Kevin B. Passi, Lukas J. Sobocinski, Cari A. Young, Bruce R. Blazar, Edwin R. Manuel

**Affiliations:** 1 Department of Immuno-Oncology, Beckman Research Institute of the City of Hope, Duarte, CA 91010, USA; nebelt@coh.org (N.D.E.); vzamloot@coh.org (V.Z.); edzuniga@coh.org (E.Z.); kpassi@coh.org (K.B.P.); lsobocinski@coh.org (L.J.S.); 2 Department of Hematology and Hematopoietic Cell Transplantation, City of Hope Comprehensive Cancer Center, Duarte, CA 91010, USA; cyoung@coh.org; 3 Department of Pediatrics, Division of Blood and Bone Marrow Transplantation, University of Minnesota Medical School, Minneapolis, MN 55455, USA; blaza001@umn.edu

**Keywords:** pancreatic ductal adenocarcinoma, targeted therapies, therapeutic resistance, tumor microenvironment, desmoplasia, collagen, collagenase, attenuated *Salmonella typhimurium*

## Abstract

**Simple Summary:**

The deposition of fibrotic tissue within pancreatic tumors acts as a physical barrier to therapeutic treatment. Collagen constitutes a large part of this barrier and serves as an ideal target to improve delivery and efficacy of anti-cancer treatments. This study characterizes a novel bacterial-based agent engineered to degrade collagens present only in pancreatic tumor tissue. Treatment using our collagen-degrading bacteria in mouse models of pancreatic cancer resulted in significant decreases in intratumoral collagen content and pro-tumor immune cell subsets, ultimately enhancing the efficacy of immunotherapy. These results support the idea that overcoming fibrosis in pancreatic cancer can dramatically improve therapeutic outcomes.

**Abstract:**

Therapeutic resistance in pancreatic ductal adenocarcinoma (PDAC) can be attributed, in part, to a dense extracellular matrix containing excessive collagen deposition. Here, we describe a novel *Salmonella typhimurium* (ST) vector expressing the bacterial collagenase *Streptomyces omiyaensis* trypsin (SOT), a serine protease known to hydrolyze collagens I and IV, which are predominantly found in PDAC. Utilizing aggressive models of PDAC, we show that ST-SOT selectively degrades intratumoral collagen leading to decreases in immunosuppressive subsets, tumor proliferation and viability. Ultimately, we found that ST-SOT treatment significantly modifies the intratumoral immune landscape to generate a microenvironment that may be more conducive to immunotherapy.

## 1. Introduction

Pancreatic ductal adenocarcinoma (PDAC) represents >90% of all pancreatic cancers and currently has a dismal five-year survival rate of 10% [1,2]. Diagnostic efforts to detect early disease are hindered due to lack of specific symptoms, advanced local tumor growth, and early metastasis, the latter of which frequently excludes surgical resection as a viable treatment [3,4]. Treatment options beyond resection are limited due to the chemoresistance of PDAC, attributed to its characteristic desmoplastic reaction that results in significant increases in collagen content compared to healthy pancreas [5,6]. Of the 28 types of collagen, collagen I (fibrillar) constitutes the majority of PDAC stroma and has been suggested to promote PDAC cell migration, proliferation, and invasion [7,8,9]. Collagen IV (non-fibrillar, mesh-like), which is additionally upregulated by tumor-associated stromal cells, is highly expressed in pancreatic tumors and has been found to promote cancer cell growth [10,11]. Pancreatic cancer cells can also produce these stromal components in vitro and in vivo, with collagen IV synthesis exceeding that of any other collagen found in PDAC [12].

High expression of collagen and other ECM components, such as hyaluronan, significantly correlates with decreased median survival in PDAC patients [13]. In particular, collagen fibers become increasingly aligned around cancerous pancreatic tissue, compared to their behavior in normal ducts, and directly correlates with worse prognosis [14]. Collagen-dense stroma and basement membranes in PDAC have been shown to contribute to rigidity, interstitial fluidic pressure, and immunosuppressive signaling that has been suggested to prevent delivery of chemotherapy and immunotherapeutic treatments and reduce immune infiltration [15,16]. This markedly inhibits the efficacy of immunotherapies such as chimeric antigen receptor T cell therapy and immune checkpoint blockade (ICB) [17,18]. Immunosuppressive properties of elevated collagen content have also been demonstrated in other solid tumor models, such as Lewis Lung Carcinoma (LLC1). Peng et al. recently demonstrated that elevated collagen in LLC1 tumors was associated with decreased effector CD8^+^ T cell infiltration and an increase in anergic CD8^+^ T cells, resulting in reduced effectiveness of PD-1/PD-L1 checkpoint blockade that was reversed upon degradation of the collagen through suppression of LOXL2, an enzyme that stabilizes and promotes collagen deposition [19]. Thus, degradation of collagen and other fibrous stromal components may improve outcomes for PDAC patients by removing physical barriers and alleviating immunosuppressive signaling to enable more effective delivery of existing anticancer agents [20].

The structure of collagen provides resistance to degradation by most proteases, however, collagenolytic proteases, or collagenases (mammalian or bacterial), can degrade collagen directly through cleavage at specific sites [21]. Although infrequently explored, bacterial collagenases, which have been identified as either metalloproteases or serine proteases [22], are able to degrade collagen with minimal recruitment or binding domains as well as recognize multiple collagen cleavage motifs [22]. These novel structures and collagenolytic mechanisms may enable improved expression in vivo as well as increased activity against collagen, ultimately enhancing direct collagen degradation compared to previously reported methods [23].

As collagens I and IV comprise a significant portion of PDAC stroma and are noted for promoting progression, proliferation, and migration of PDAC cells, they are ideal targets for PDAC stromal degradation; however, collagens I and IV are abundant in normal tissues [24], similar to other elements of tumor stroma. Previously we have shown that attenuated *Salmonella typhimurium* (ST) engineered to express bacterial hyaluronidase could effectively degrade hyaluronan in PDAC tumors to improve efficacy of gemcitabine treatment without off-target degradation in tissues high in hyaluronan such as the skin or joints [25]. This is due to preferential colonization of tumors by attenuated ST compared to healthy tissues at a ratio >6000:1 [26,27]. In order to efficiently and preferentially degrade tumor-associated collagen in a similar manner, we selected *Streptomyces omiyaensis* trypsin (SOT), a small-molecular weight bacterial collagenase that has specificity for collagens I and IV [28], for inducible expression in ST (ST-SOT) with the overall goal of increasing delivery of anti-cancer medications. We employed ST-SOT in preclinical models of PDAC that overexpress the major collagen types I and IV and observed tumor-specific collagen degradation with resultant decreases in immune checkpoint marker expression and tumor growth. The ability of ST-SOT to simultaneously reduce tumor fibrosis and immunosuppressive subsets may lend itself well to combination treatment with currently available immunotherapies. This approach to tackling tumor desmoplasia meets the requisites for a safe, inexpensive, and effective remedy that may improve treatment outcomes for patients with stroma-rich, therapy-resistant cancers such as PDAC.

## 2. Results

### 2.1. Inducible SOT Expression in Attenuated Salmonella Typhimurium (ST-SOT)

**Figure 1:**
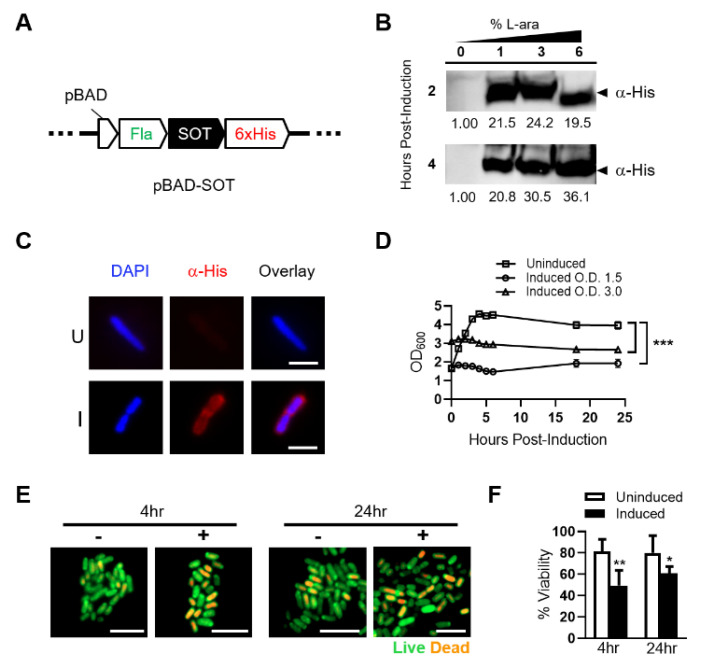
Expression, subcellular localization and toxicity of SOT in attenuated ST. (**A**) Schematic of inducible pBAD construct (pBAD-SOT) integrating ampicillin resistance with N-terminal flagellin (Fla) fusion and C-terminal 6XHis tag fusion to SOT for downstream analyses. (**B**) Attenuated ST transformed with the pBAD-SOT construct was cultured in Luria Broth (LB) containing 0% (uninduced) or 1–6% (induced) L-arabinose for up to 4 h at 37 °C. Bacterial cell lysates from ~5 × 10^7^ colony forming units (CFUs) at each time point for each L-arabinose concentration were run on a 4–20% polyacrylamide gradient gel and subjected to Western blot analysis against a His-tag fused to the C-terminus of SOT (α-His). Predicted SOT fusion size ~31 kDa (arrow). Band densitometries are shown under blots relative to uninduced. (**C**) ST-SOT was cultured in LB media containing 2% L-arabinose for 1 h and then immunostained (α-His) to determine subcellular localization of SOT (red). Nuclei are stained with DAPI (blue). Representative images shown. Scale bar = 10 µm. (**D**) Growth curve of ST-SOT post-induction. Optical density readings (OD_600_) for uninduced and induced (2% L-ara) ST-SOT cultures were measured over 24 h. Uninduced and induced cultures were done in triplicate and error bars represent standard error of the mean. Growth curves of uninduced and induced are compared. *** *p* < 0.001, two-way ANOVA. (**E**) ST-SOT under uninduced and induced conditions were stained at indicated time points with acridine orange (live, green) and ethidium bromide (dead, orange) and imaged by fluorescence microscopy at 100× magnification. Scale bar = 5 µm. (**F**) Percent viability of ST-SOT under uninduced and induced conditions at indicated time points based on live/dead staining from six random fields at 100× magnification. * *p* < 0.05, ** *p* < 0.01, *t*-test. Experiments performed ≥2 times.

We utilized the P_BAD_ promoter of the araBAD (arabinose) operon to control SOT expression in order to prevent toxic effects associated with foreign protein expression in ST (Figure 1A) [29]. For the best possible expression, we also synthesized the SOT sequence with codons most utilized by ST [30]. Additionally, we fused the ST flagellin signal sequence to the amino terminus of SOT to facilitate greater surface display and incorporated a C-terminal His-tag for downstream detection. A single plasmid preparation of pBAD-SOT was used for electroporation into the clinical ST strain YS1646, also known as VNP20009 [31]. A positive clone, identified by colony PCR, was cultured under uninduced (media only) and induced (increasing L-arabinose) conditions, followed by Western blot (WB) of pellet lysates. WB revealed robust expression of His-tagged SOT at the correct molecular weight (~31 kD) at all percentages of L-arabinose as well as tight regulation of protein expression under uninduced and induced conditions (Figure 1B and Figure S1).

We determined the subcellular location of SOT expressed by ST using a His-tag fused to the carboxy-terminus of the protein (Figure 1C). Immunofluorescent staining revealed subcellular localization of His-tagged SOT outside of genomic DNA staining (cytoplasm), suggesting that it is located at the outer membrane of the bacterium. Overall, these results confirm that expression of SOT is tightly regulated and that its subcellular location is restricted to the outer membrane of ST.

Successful expression of functional SOT may have detrimental effects on ST growth kinetics. Thus, we performed optical density (O.D.) readings following induction to determine growth kinetics over a 24-h period. ST-SOT reached a maximum O.D. within 6 h that was maintained through 24 h (Figure 1D). Following induction, there was no observable increase or decrease from initial O.D., suggesting that SOT expression attenuates replication without causing immediate ST loss. To further investigate bacterial viability after induction, we performed live/dead staining using a mixture of acridine orange (AO) and ethidium bromide (EB), respectively, at 4 and 24 h under uninduced and induced conditions [32,33]. As shown in Figure 1E, the percentage of live bacteria 4 h after induction was not significantly different through 24 h but was significantly different from percent viability observed for uninduced ST-SOT. Overall, these results confirm robust expression of SOT by ST that is associated with fitness cost to ST, thus emphasizing the importance of using an inducible system to allow for initial expansion of ST-SOT and robust SOT expression for subsequent in vitro and in vivo functional studies.

### 2.2. ST-SOT Hydrolyzes Major Collagen Substrates

**Figure 2:**
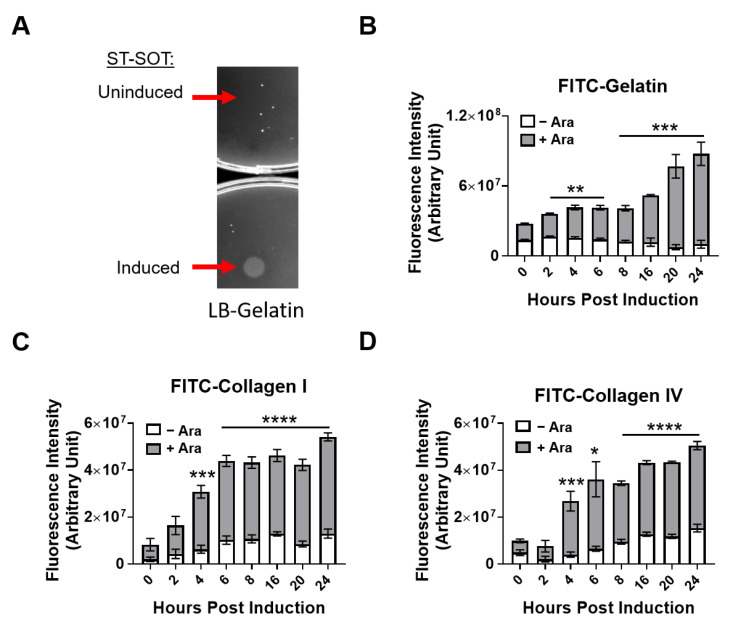
Hydrolytic collagenase activity of ST-SOT towards various substrates. (**A**) Uninduced or induced (2% L-arabinose) ST-SOT was incubated on LB-gelatin plates overnight (16 h) at 37 °C. Hydrolysis of gelatin in LB agar media is observed as opaque areas on LB-gelatin plates. Arrows indicate areas where uninduced or induced ST-SOT were spotted onto the plate. (**B**) Hydrolysis reactions were performed using uninduced or induced ST-SOT co-incubated with FITC-conjugated pig skin gelatin, bovine skin collagen type I (**C**) or human placenta collagen type IV (**D**) in 50 mM Tris-HCl (pH 8.0) containing 10 mM CaCl_2_ at 37 °C. Enzyme activity was measured by monitoring fluorescence (FITC) (ex: 495 nm, em: 519 nm). Data are expressed as mean ± error of mean of three independent experiments. * *p* < 0.05, ** *p* < 0.01, *** *p* < 0.001, **** *p* < 0.0001, *t*-test.

To measure the enzymatic activity of ST-SOT on known substrates we used gelatin-agar plates and fluorescently-labeled substrate assays [34,35]. Hydrolysis of gelatin on gelatin-agar plates results in the formation of a visible white precipitate [36]. For these studies, we cultured ST-SOT under induced (2% L-arabinose) or uninduced conditions and then pipetted 5 µL of culture (approximately 1 × 10^8^ colony forming units (CFUs)) onto gelatin-agar plates overnight. An area of hydrolysis was observed for ST-SOT under induced conditions that was limited to the size of the colony, suggesting that SOT is anchored to the outer membrane and not secreted (Figure 2A). The ability of ST-SOT to hydrolyze gelatin was further confirmed using FITC-labeled gelatin, which is converted into fluorescent peptides when hydrolyzed. As shown in Figure 2B, induced ST-SOT (+Ara) caused significant increases in fluorescence intensity, compared to uninduced control (-Ara), within 4 h and continued to increase through 24 h. These results are the first to suggest that SOT expressed by ST has sufficient functional activity to hydrolyze the less-complex gelatin. We next determined whether ST-SOT could hydrolyze the major collagen types found in PDAC, namely I and IV. Indeed, induced ST-SOT also caused significant increases in fluorescence intensity when co-incubated with FITC-labeled collagen I or IV (Figure 2C,D), further confirming that SOT expressed by ST exhibits collagenolytic function. Additionally, we observed no significant change in fluorescence intensity when FITC-labeled substrates were co-incubated with uninduced ST-SOT, emphasizing tight regulation by the inducible pBAD promoter system.

### 2.3. In Vivo Depletion of Collagens by ST-SOT Is Restricted to PDAC Tumor Tissue and Augments ST Diffusion

**Figure 3:**
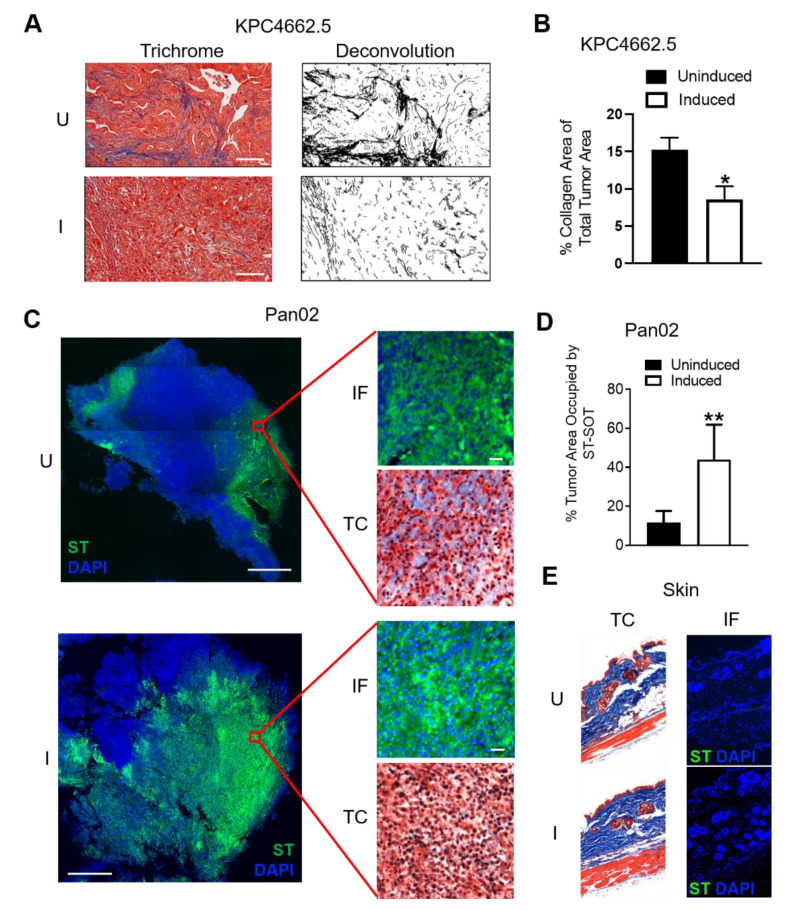
Induction of ST-SOT leads to intratumoral CN depletion and enhanced ST diffusion in vivo. (**A**) C57BL/6 mice bearing orthotopic KPC4662.5 tumors (>150 mm^3^) were administered 5 × 10^6^ colony-forming units (CFU) of ST-SOT by intravenous (i.v.) injection. Forty-eight hours later, groups were intraperitoneally (i.p.) administered PBS (uninduced) or 250 mg L-arabinose (induced) and then euthanized 48 h post-induction. Tumor sections were then subjected to Masson’s trichrome staining (representative images shown, scale bar = 50 µm). Deconvolution of collagen (black) was performed using ImageJ and percent collagen area present within total tumor section (**B**) was determined. * *p* < 0.05, *t*-test. (**C**) C57BL/6 mice bearing subcutaneous Pan02 tumors (>150 mm^3^) were i.v. administered 5 × 10^6^ cfu ST-SOT and euthanized 48 h after i.p. injection of PBS (uninduced, U) or L-arabinose (induced, I). Serial tumor sections were subjected to immunofluorescence (IF) staining to detect ST-SOT (ST), as well as trichrome (TC) staining (TC). Representative images shown, scale bar = 200 µm (inset scale bar = 30 µm). Percent area of ST occupying total tumor area (approximated by DAPI staining) (**D**) was determined using Image One software. ** *p* < 0.01, *t*-test. (**E**) IF and TC staining were performed on skin from Pan02-bearing mice receiving uninduced or induced ST-SOT treatment. Representative images shown.

We next determined the ability of ST-SOT to colonize and deplete collagen in orthotopic (o.t.) Kras^G12D^p53^R172H^Cre^Pdx1^ (KPC) 4662.5 and subcutaneous (s.c.) Pan02 tumors when delivered intravenously into wildtype C57BL/6 mice [37,38]. We first verified that the YS1646 vector used in the construction of ST-SOT was capable of colonizing o.t. and s.c. tumors by using a constitutive bacterial reporter construct encoding the bioluminescent LUX operon [39]. When 5 × 10^6^ CFU recombinant YS1646 encoding LUX (ST-LUX) was injected intravenously (i.v.) into C57BL/6 mice bearing o.t. KPC4662.5 or s.c. Pan02 tumors (>150 mm^3^), we observed bioluminescence localized to the area of the tumor, which was typically detected by day ~2 and disappeared by day ~7 (data not shown [40]). To further verify tumor-specific colonization by ST-LUX, we examined o.t. KPC4662.5 tumors, spleen, and liver [27] 48 h following i.v. injection and measured bioluminescence in each tissue type. Indeed, ST-LUX was highly concentrated in o.t. tumor tissue and completely absent in both spleen and liver (Figure S2A). These results suggest that the YS1646 vector is capable of colonizing o.t. and s.c. tumors after systemic administration and that peak tumor colonization is achieved by day 2, which represents an ideal time point for SOT induction. Thus, C57BL/6 mice with o.t. KPC4662.5 tumors (>150 mm^3^) were i.v. administered 5 × 10^6^ CFUs of ST-SOT and then induced 2 days later by a single intraperitoneal (i.p.) injection of 250 mg of L-arabinose per mouse. KPC4662.5 tumors were excised 48 h after induction, sectioned, and stained using Masson’s trichrome. As shown in Figure 3A, tumors from mice treated with ST-SOT under inducing conditions showed significantly reduced collagen content compared to collagen content in tumors from uninduced mice (Figure 3B, *p* < 0.05). Similarly, s.c. Pan02 tumors were observed to have decreased collagen content in regions colonized by induced ST-SOT, detected by immunofluorescence (Figure 3C), which was also associated with significantly greater ST diffusion throughout tumor tissue (Figure 3D and Figure S2B). ST colonization and reduction in collagen content were not observed in healthy tissue such as the skin (Figure 3E) and joints (Figure S2C) under inducing conditions. These data suggest that ST-SOT effectively colonizes PDAC tissue, whether located o.t. or s.c., and degrades tumor-associated collagens that allow for greater influx of large molecular weight objects, such as ST, from the bloodstream.

### 2.4. ST-SOT Treatment Reduces Frequency of Suppressive Intratumoral Immune Subsets

**Figure 4:**
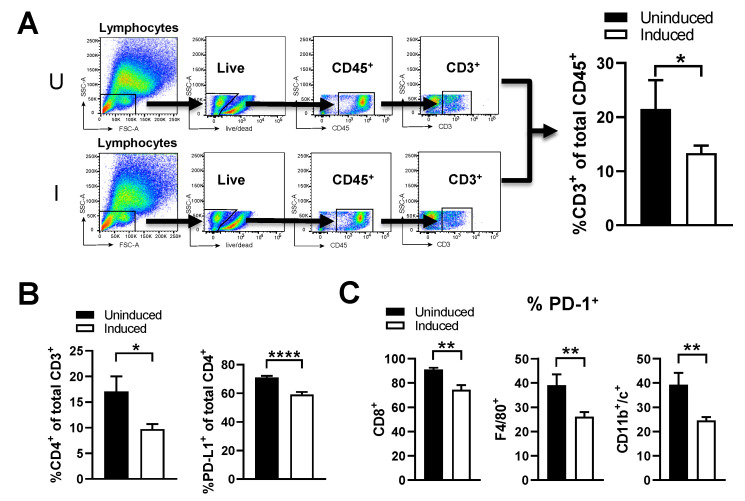
ST-SOT treatment reduces intratumoral frequency of suppressive immune subsets. Pan02 tumor-bearing mice were treated with ST-SOT under inducing conditions (I) or uninduced (U). Forty-eight hours later, mice were euthanized and tumor homogenates were subjected to flow cytometry to evaluate intratumoral immune phenotypes. (**A**) Initial gating strategy on CD3^+^ cells in tumors from uninduced and induced treatment groups (*n* = 5). Additional analyses of CD3^+^ immune subsets were performed to quantify frequency of (**B**) CD4^+^ and PD-L1^+^CD4^+^ cells. (**C**) Percent of PD-1^+^ immune subsets was determined for CD8^+^ T cells, macrophages (F4/80^+^), and dendritic cells (CD11b^+^CD11c^+^). * *p* < 0.05, ** *p* < 0.01, **** *p* < 0.0001, *t*-test. Experiments performed ≥2 times.

Significantly high collagen content is known to increase intratumoral frequencies of suppressive immune subsets that blunt anti-tumor responses [19,41]. To determine the effects of reducing collagen content in PDAC tissue, we evaluated intratumoral immune subsets following ST-SOT treatment by flow cytometry in mice bearing Pan02 tumors. From our initial gating of total CD45^+^ cells, we immediately observed dramatic decreases in CD3^+^ T cells following induction (Figure 4A, *p* < 0.05). Within this CD3^+^ population, CD4^+^ T cells were significantly decreased in induced mice (*p* < 0.05), compared to uninduced, specifically those co-expressing PD-L1^+^ (Figure 4B, *p* < 0.0001), which are known to induce apoptosis or anergy in effector T-cells [42]. Moreover, induced ST-SOT treatment was shown to decrease the intratumoral frequency of PD-1-expressing CD8^+^ T cells, macrophages (F4/80^+^CD11b^+^), and dendritic cells (CD11b^+^CD11c^+^) (Figure 4C, *p* < 0.001). Modulation of these specific immune subsets by collagen is consistent with previous studies [19,43]. Overall, these results suggest that ST-SOT treatment can modulate the intratumoral landscape to produce a microenvironment more conducive to immunotherapy.

### 2.5. ST-SOT Treatment Effectively Controls Tumor Growth

**Figure 5:**
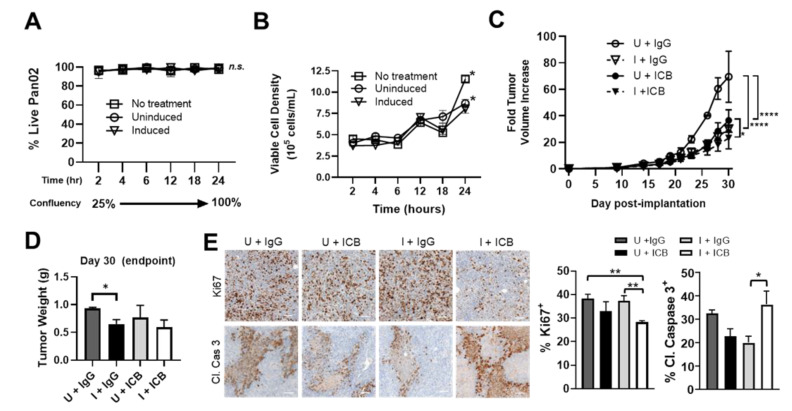
ST-SOT augments the anti-tumor effects of immune checkpoint blockade (ICB) treatment and is associated with a more permissive tumor immune microenvironment. (**A**) The direct effects of ST-SOT on tumor cell growth was determined through co-incubation with Pan02 cells at a confluency of 25% in antibiotic-free culture medium and using a starting multiplicity of infection = 50. Percent cell viability was determined by trypan blue staining at various time points in uninfected culture (no treatment) or post-infection under inducing (2% L-arabinose) or non-inducing conditions for 24 h. Kinetics of tumor cell growth (cells/mL) (**B**) were also performed simultaneously over the 24 h co-incubation period. * *p* < 0.05, two-way ANOVA. (**C**) C57BL/6 mice bearing s.c. Pan02 tumors (average 100 mm^3^, *n* = 3–5) were i.v. administered ST-SOT (5 × 10^6^ CFU) and then i.p. administered 250 mg L-arabinose (induced, I) or PBS (uninduced, U). At induction/PBS, mice were i.p. administered immune checkpoint blockade (ICB) antibodies (anti-PD-1 (200 µg) + anti-CTLA-4 (75 µg)) or control IgG antibody, with maintenance treatments every 3 days at a reduced dose. Fold tumor volume change was determined by dividing tumor volume at a given time point by the initial tumor volume when ST-SOT was first administered. * *p* < 0.05, **** *p* < 0.0001, two-way ANOVA. (**D**) Tumor weights for each treatment group (*n* = 3–5) were measured at endpoint (Day 30). * *p* < 0.05, *t*-test. (**E**) Tumors excised at endpoint were processed for sectioning and analyzed by IHC (brown staining) with anti-cleaved caspase 3 antibody (Cl. Caspase 3) or anti-Ki67 antibody. Nuclei were counterstained with hematoxylin (blue) (representative images shown, scale bar = 50 µm). Bar graphs represent percentage of positive cells out of total nuclei. * *p* < 0.05, ** *p* < 0.01, *t*-test.

To determine the therapeutic effects of ST-SOT treatment on tumor cell growth, we performed a series of in vitro and in vivo studies. Tumor growth control by ST alone is dependent on the presence of innate immune subsets, such as neutrophils and macrophages, that exert both bacteriocidal and cytolytic activity [44,45]. However, engineered strains of attenuated ST may also exert anti-tumor activity when cultured directly with tumor cells that is independent of media depletion or pH change, which can be observed in the first 24 h of co-incubation [46]. Whereas co-culturing with ST-SOT did not affect the overall viability of Pan02 tumor cells under inducing conditions (Figure 5A), a significant decrease in growth kinetics was observed over 24 h compared to tumor cells cultured under uninduced conditions or only L-arabinose (no ST treatment) (Figure 5B). These results suggest that SOT expressed by ST may delay tumor cell growth in culture through degradation of collagens required for autocrine signaling involved in proliferation and survival [5,11,47].

Based on the ability of ST-SOT treatment to reduce intratumoral suppressive immune subsets, we evaluated combination treatment with immune checkpoint blockade (ICB) antibodies against PD-1 and CTLA-4 [48,49]. As shown in Figure 5C, ICB with ST-SOT under uninduced conditions (U+ICB) or ST-SOT with IgG under induced conditions (I+IgG) resulted in significant tumor growth control (*p* < 0.0001) compared to uninduced ST-SOT plus IgG control (U+IgG) treatment. However, ICB plus ST-SOT under induced conditions (I+ICB) resulted in the greatest tumor growth control (*p* < 0.05). Whereas I+ICB reduced tumor weights at endpoint, only I+IgG treatment reached significance (*p* < 0.05) when compared to U+IgG-treated groups (Figure 5D). Analysis of tumors at endpoint to evaluate proliferation by Ki67 expression or apoptosis by cleaved caspase-3 expression revealed significant decreases in Ki67-positive cells in the I+ICB group compared to U+IgG or I+IgG (*p* < 0.01), and significantly greater expression of cleaved caspase-3 compared to the I+IgG group but not U+IgG and U+ICB groups (Figure 5E, *p* < 0.05). Of note, larger tumors, such as those from the U+IgG group may generally show increased expression of cleaved caspase-3 in tumor centers due to hypoxia and lack of blood flow, compared to smaller tumors. Thus, the increased cleaved caspase 3 in the I+ICB group compared to similarly sized (small) tumors (I+IgG) is likely due to direct action of the treatment. Taken together, these data indicate that tumors from the I+ICB group demonstrate significantly decreased cell proliferation and/or increased apoptosis compared to tumors from all other groups.

### 2.6. Combination ST-SOT with ICB Therapy Prevents Increases in Collagen Deposition and Checkpoint Expression Concurrent with ICB Therapy Alone

**Figure 6:**
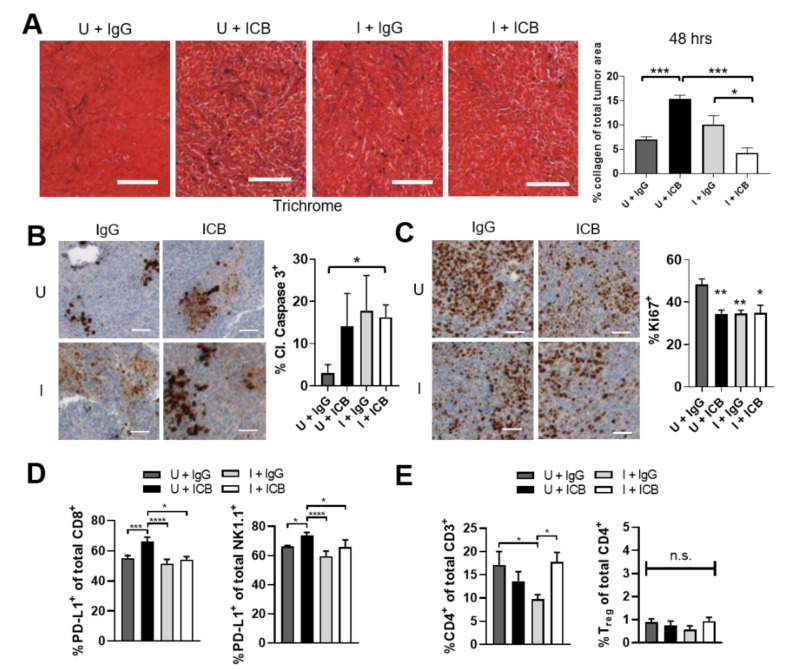
ST-SOT with immune checkpoint blockade (ICB) treatment augments tumor cell apoptosis, prevents increased expression of PD-L1 associated with ICB, and preserves CD4 populations associated with collagen degradation. C57BL/6 mice bearing s.c. Pan02 tumors (average 100 mm^3^, *n* = 3–5) were i.v. administered ST-SOT (5 × 10^6^ CFU) and then i.p. administered 250 mg L-arabinose (induced, I) or PBS (uninduced, U). At induction/PBS, mice were i.p. administered immune checkpoint blockade (ICB) antibodies (anti-PD-1 (200 µg) + anti-CTLA-4 (75 µg)) or control IgG antibody. Forty-eight hours later tumors were excised and processed for sectioning and flow cytometry. (**A**) Tumors were sectioned, stained using Masson’s trichrome method, and analyzed for collagen density (representative images shown, scale bar = 100 µm). Bar graph represents whole tumor quantification of collagen stain out of total tumor area (*n* = 3). * *p <* 0.05, *** *p <* 0.001, *t*-test. Sectioned tumors were stained for IHC with anti-cleaved caspase 3 antibody (brown) (**B**) or anti-Ki67 antibody (brown) (**C**). Nuclei were counterstained with hematoxylin (blue) (representative images are shown, scale bar = 50 µm). Bar graphs represent whole tumor quantification of positive cells out of total nuclei. * *p <* 0.05, ** *p <* 0.01, *t*-test. (**D**) Tumors processed for flow cytometry were analyzed for PD-L1-expressing immune subsets and (**E**) CD4^+^CD25^+^FoxP3^+^ cells (Tregs). * *p <* 0.05, *** *p <* 0.001, **** *p <* 0.0001, *t*-test.

In order to determine the mechanistic effects of combination ST-SOT treatment with ICB therapy in Pan02 tumors, early analyses of collagen content and immune subsets immediately after a single combination treatment (48 h) were performed. Analysis of intratumoral collagen content revealed an approximate doubling of collagen in the U+ICB compared to the U+IgG group, consistent with dual anti-PD-1, anti-CTLA-4 blockade [50] (Figure 6A, *p* < 0.001). However, I+ICB prevented this increase and showed a significant decrease compared to induction alone (I+IgG) (Figure 6A, *p* < 0.05). The collagen content of tumors in the I+IgG group was not significantly different from tumors in the U+IgG group, possibly indicating a longer time for clearance of degraded collagen when ICB is not present. Assessment of tumor cell apoptosis also demonstrated an immediate response to the I+ICB combination, with this treatment showing the only significant upregulation in cleaved caspase-3 compared to the U+IgG group (Figure 6B, *p* < 0.05). Tumor cell proliferation, as measured by Ki67, was also significantly decreased in the I+ICB group compared to U+IgG- treated mice (*p* < 0.05), as was tumor cell proliferation in the U+ICB and I+IgG groups (Figure 6C, *p* < 0.01), in agreement with tumor growth control observed by these treatment groups compared to U+IgG treatment in Figure 5C. Analysis of immune subsets revealed a significant upregulation of PD-L1-expressing CD8^+^ and NK cells following ICB treatment (U+ICB) compared to IgG control-treated mice (U+IgG) (Figure 6D, *p* < 0.001 and *p* < 0.05, respectively). Interestingly, groups receiving ST-SOT treatment under induced conditions blunted increases in both PD-L1-expressing subsets following ICB treatment (I+ICB vs. U+ICB, *p* < 0.05). While we did observe significant increases in CD4^+^ T cells in I+ICB-treated groups compared to I+IgG control (Figure 6E, *p* < 0.05), these CD4^+^ T cells were not CD25^+^FoxP3^+^ (i.e., Tregs). Ultimately, ST-SOT treatment may enhance the efficacy of ICB therapy by reducing stromal collagen and the prevalence of suppressive subsets pre- and post-ICB therapy.

## 3. Discussion

Therapeutic resistance continues to be a major factor contributing to poor survival rates in pancreatic cancer. While many anticancer drugs prove potent in vitro, many have failed in the clinic because they are unable to penetrate tumor tissue in sufficient amounts to be therapeutic while also averting adverse effects. Eliminating tumor ECM components, such as collagen, is hypothesized to improve anticancer drug delivery by decreasing solid stresses and normalizing tissue vasculature [47]. Various preclinical studies utilizing collagen-targeted therapies have demonstrated promising outcomes but remain controversial due to the abundance of collagens throughout the body and lack of tumor-specificity associated with these approaches [20,51,52]. In addition, concerns remain about increasing metastasis of tumor cells released from the matrix after ECM degradation. In previous studies, inhibition of stromal deposition by genetic ablation or pharmacologic inhibition of Sonic hedgehog in an autochthonous PDAC model increased tumor growth and metastasis [53]. In clinical trials, pharmacologic inhibition of hedgehog did not increase the efficacy of chemotherapy in patients with metastatic PDAC despite increasing vessel dilation after stromal depletion [54]. However, direct enzymatic degradation of stromal components including hyaluronan and collagen improves chemotherapeutic efficacy and decreases metastasis in PDAC models [55,56].

To achieve tumor-targeted, enzymatic collagen degradation, we engineered ST that preferentially colonizes tumors to express the collagen-degrading SOT enzyme. After treatment we find that ST is concentrated in tumors with no ST signal found in other major organs such as heart and lungs (by whole mouse imaging) or collagen-rich tissues such as skin and bone (by immunofluorescence) [57,58]. In addition, induced SOT enzyme was found to anchor to the bacterial surface, minimizing potential for secretion into systemic circulation. Thus, this system prevents off-target collagen degradation, reducing possible toxic side effects.

We found that induction of tumor-colonizing ST-SOT resulted in significant reduction of collagen content and greater bacterial diffusion. Interestingly, significant reduction of suppressive intratumoral subsets was observed following ST-SOT treatment alone and subsequent to combination treatment with ICB. Ultimately, ST-SOT pre-treatment facilitated greater tumor growth control following ICB treatment compared to ICB alone. These studies are among the first to describe a tumor-targeted strategy to degrade major collagens within PDAC tissue leading to significant improvement in immunotherapeutic efficacy.

Previous studies have shown that a high-density collagen matrix within tumors is associated with decreased cytotoxic T cell abundance and increased regulatory T cell infiltration [59,60,61]. In line with this, collagen molecules are known to act as a ligand for the inhibitory leukocyte-associated immunoglobulin-like receptor 1 (LAIR-1), which is encoded by NK, T and B cells [62,63]. Signaling through this receptor induces T cell exhaustion and increases resistance to ICB [19,64]. Collagen degradation after ST-SOT pre-treatment may interfere with this ligand–receptor interaction, sensitizing tumors to ICB treatment, consistent with increased sensitivity to ICB in tumors with low collagen deposition [19]. Interestingly, in this study we observed a 2-fold increase in collagen content by 48 h after ICB treatment, which has been observed previously in a pre-clinical colon cancer model [50]. The combination of ICB with induction of ST-SOT in our model prevented this increase and synergized to decrease collagen in the stroma greater than induction with IgG, possibly implying that IgG treatment increases collagen somewhat similarly to ICB treatment. Regardless, increased collagen after an initial treatment with ICB may decrease the diffusion of further ICB treatments to the tumor, consistent with a more recently described mechanism of acquired resistance to ICB [19].

Fifty percent of PDAC diagnoses occur at late-stage, and nearly all histopathologic analyses of primary tumor and metastases show extensive desmoplasia [13]. However, the abundance of each ECM component contributing to desmoplasia is not always uniform. Collagen imparts mechanical stresses that act in concert with other major ECM components, such as hyaluronan, to limit tumor perfusion [65,66]. Whereas an overabundance of hyaluronan contributes to increased interstitial fluidic pressure leading to vessel compression, collagen fibers impart rigidity to maintain tissue-level compression that prevents stromal collapse and, in turn, vessel decompression. Thus, strategies employing only individual depletion of hyaluronan or collagen, even if done effectively, may not result in maximum tumor permeability. A combinatorial strategy to target both hyaluronan and collagen simultaneously not only presents an approach to maximize drug delivery in PDAC with broader applicability, but also presents greater risk of adverse events. To overcome this possibility, we have also engineered recombinant ST expressing bacterial hyaluronidase (bHs-ST) [25]. A mixture containing both ST-SOT and bHs-ST is predicted to simultaneously deplete tumor-associated collagen and hyaluronan with minimal off-tumor toxicity. This is in contrast to previously tested small-molecule inhibitors, which inadvertently increase tumor aggressiveness and metastasis as a result of targeting signaling pathways involved in collagen and hyaluronan synthesis [67,68]. Our ST-based platform allows for safe targeting of both components for the first time and will require additional studies to determine the added benefits of dual versus single depletions as well as effects on metastatic potential, which remains a major concern with ECM remodeling. In addition to PDAC, our findings may have broader application to other desmoplastic tumor types such as those originating in the breast and lung [69].

## 4. Materials and Methods

### 4.1. Animals and Cell Lines

C57BL/6 mice were obtained from breeding colonies housed at the City of Hope (COH) Animal Research Center. The Pan02 cell line was a kind gift from Dr. DC. Linehan, Washington University School of Medicine [70] and the KPC4662.5 cell line was a kind gift from Dr. Robert Vonderheide, University of Pennsylvania [37]. Cells were maintained in DMEM media containing 10% FBS, 2 mM L-glutamine and pen/strep. KPC4662.5 cells, prior to orthotopic implantation, were passaged ≤5 times and maintained at ≤80% confluency in DMEM containing 10% FBS, 2 mM L-glutamine and pen/strep.

### 4.2. ST Strains and Generation of ST-SOT

YS1646 (ST) was obtained from ATCC^®^ (202165^TM^) and cultured in modified LB media lacking NaCl (LB-0). The SOT amino acid sequence (GenBank Accession no. AB362837.1) was used to synthesize a codon-optimized cDNA fused to Fla (N-terminus) and 6XHis-tag (C-terminus) (Biomatik, Wilmington, DE, USA), which was inserted into a pBAD bacterial expression vector (kind gift from Michael Davidson, Addgene #54762) to generate pBAD-SOT. ST-LUX was generated using the pAKlux2 plasmid (pAKlux2 was a gift from Attila Karsi, Addgene #14080). Plasmids were electroporated into YS1646 and spread onto LB-0 plates containing 100 µg/mL ampicillin and incubated overnight at 37 °C. Positive clones were identified by colony PCR and restriction digest.

### 4.3. Bacterial Growth, Viability, and Analysis of SOT Expression

Clones of ST electroporated with pBAD-SOT were cultured in media with or without 2% (*w/v*) L-arabinose at 37 °C for 1–24 h. Growth kinetics were monitored through analysis of absorbance at 600 nm (Genesys 30, Thermo Scientific; Waltham, MA, USA) every 1–2 h. SOT expression was detected in bacterial lysates by Western blot using an anti-6XHis antibody (Proteintech; Rosemont, IL, USA) and localization of SOT was detected by immunofluorescence using a primary monoclonal mouse antibody against ST (Clone 1E6, Thermofisher; Waltham, MA, USA). Western blot band densitometry was performed on original western tiffs using ImageJ (NIH).

### 4.4. Gelatin-LB Plate and Fluorometric Substrate Assays

ST-SOT was cultured under uninduced or induced (2% L-arabinose) conditions for 1 h at 37 °C and then pipetted onto 1% gelatin-agar plates (1 × 10^8^ CFU in 100 µL) and incubated overnight at 37 °C [71]. To measure enzymatic activity of ST-SOT, substrates consisting of bovine skin collagen type I, human placenta collagen type IV, and pig skin gelatin conjugated to FITC (Thermofisher; Waltham, MA, USA) were used. The reaction was started by the addition of ST-SOT under uninduced or induced (2% L-arabinose) conditions and subsequently monitored to assess the increase in fluorescence intensity over 24 h using an iBright FL1500 Imaging System (Thermofisher; Waltham, MA, USA).

### 4.5. Orthotopic and Subcutaneous Tumor Implantation

Orthotopic implantation of KPC4662.5 cells into the mouse pancreas was achieved using previously published methods [72]. Briefly, small incisions were made through the skin and peritoneum. The pancreas was identified and, using a 27 gauge needle, approximately 2 × 10^5^ KPC4662.5 cells in a volume of 50 µL (10% matrigel, BD Biosciences, San Jose, CA, USA) were injected into the body. The pancreas was reinserted, and incisions were closed using absorbable sutures and staples. Analgesics were administered pre- and post-surgery. Pan02 cells (2 × 10^5^) were implanted subcutaneously above the right flank in a total volume of 50 µL.

### 4.6. Quantification of Collagen Degradation

Paraffin-embedded tumors were sectioned and stained using Masson’s Trichrome. Whole tumors were imaged using the Zeiss Axio Observer II microscope (Carl Zeiss Inc.; White Plains, NY, USA) and trichrome images were color deconvoluted using the “Masson’s Trichrome” setting in ImageJ (U. S. National Institutes of Health; Bethesda, MD, USA). Thresholds were set for individual channels just under background and quantified using the “analyze particles” option under “measure.” Total collagen area (blue channel) was divided by the addition of collagen area plus total cytoplasm area (red channel) to obtain the percentage of collagen.

### 4.7. Immunohistochemistry

IHC was performed using a Ventana Discovery Ultra IHC autostainer (Roche Diagnostics; Indianapolis, IN, USA) according to manufacturer’s protocols. Briefly, FFPE samples were sectioned (5 μm) and transferred to glass slides. After deparaffinizing, slides were incubated with primary antibodies, followed by DAB detection staining (Ventana Medical Systems; Santa Clara, CA, USA). The slides were counterstained with hematoxylin and cover-slipped. Antibodies used: cleaved caspase 3 and Ki67 (Cell Signaling Technologies; Danvers, MA, USA). DAB staining out of total nuclei per field was done using ImageJ (NIH). DAB and hematoxylin channels were separated using color deconvolution (H-DAB preset), and thresholds were set to cover positive staining area for DAB (brown) and positive staining nuclei for hematoxylin (blue) just under background. DAB-positive nuclei were quantified by dividing DAB threshold area by DAB threshold area plus hematoxylin threshold area.

### 4.8. Flow Cytometry

Live cells (~10^6^ cells) were counted using trypan blue and stained with a viability dye (eBiosciences 65-0866-14, Thermo Fisher Scientific; Waltham, MA, USA) for 30 min at 4 °C. Cells were washed (PBS with 0.05% sodium azide and 1% FBS) and stained with surface antibodies for 40 min at 4 °C. Cells were washed, fixed in flow buffer with 1% PFA, and then filtered using a 40 μm mesh strainer/tube (BD Biosciences; San Jose, CA, USA). Flow cytometry was performed on the BD FACSCelesta cytometer and data was analyzed using FlowJo Version 10 (Becton, Dickinson & Co.; Franklin Lakes, NJ, USA). Flow cytometry antibodies used from BD Biosciences (San Jose, CA, USA): CD45-PerCPCy5.5 (550994), CD8-APC-R700 (564983), CD4-APC-H7 (560246), Ly6G-BV605 (563005), Ly6C-FITC (561085), CD11b-APC (561690), PD-1-BV421 (562584), CTLA-4-PE (553720), CD45-APC-R700 (565478), CD3-PerCPCy5.5 (551071), NK1.1-BV650 (564143), CD11c-BV605 (563057), CD45-BV650 (563410), CD4-BV786 (563331), CD25-PerCPCy5.5 (551071), CD11c-APC-R700 (565871), and IFN-gamma-APC (554413). Flow cytometry antibodies used from eBiosciences (Thermo Fisher Scientific; Waltham, MA, USA): F4/80-Super Bright 780 (78-4801-82), PD-L1-Super Bright 645 (64-5982-82), PD-L1-Super Bright 780 (78-5982-80), and FoxP3-APC (17-5773-82). For intracellular staining against CTLA-4, cells were permeabilized using the Cytofix/Cytoperm Plus kit (555028, BD Biosciences; San Jose, CA, USA). For intracellular staining against FoxP3, cells were permeabilized using the FoxP3/transcription factor staining buffer set from eBiosciences (00-5523-00, Thermo Fisher Scientific; Waltham, MA, USA).

### 4.9. Tumor Growth Measurements and Treatment

For the Pan02 subcutaneous tumor model, tumors were allowed to grow for two weeks until reaching an average volume of 100 mm^3^. At this time, all mice were treated with 2.5 × 10^6^ CFU ST-SOT intravenously two days in a row. Three days after the first treatment, mice were randomized into four groups: (1) ST-SOT uninduced + IgG (*n* = 3), (2) ST-SOT uninduced + ICB (*n* = 4), (3) ST-SOT induced + IgG (*n* = 4), and (4) ST-SOT induced + ICB (*n* = 4). Induced groups received 250 mg L-Arabinose via intraperitoneal injection while uninduced groups received an equivalent volume of PBS. IgG groups received 200 µg Armenian Hamster IgG with 75 µg Syrian Hamster IgG (BioXCell; Lebanon, NH, USA) and ICB groups received 200 µg anti-PD-1 (J43) (gift from Dr. Blazar) with 75 µg anti-CTLA-4 (9H10) (BioXCell; Lebanon, NH, USA). Maintenance doses of IgG or ICB were given at reduced amounts (100 µg anti-PD-1/IgG and 40 µg anti-CTLA-4/IgG) every 3 days until the end of the experiment. Tumor volumes were measured thrice weekly using digital calipers. Tumor growth is represented as fold change in growth compared to volume at initial ST-SOT treatment.

### 4.10. Statistics

Prism software by GraphPad (V8) (San Diego, CA, USA) was used for statistical calculations. *T*-test and two-way ANOVA followed by Tukey’s multiple comparisons test were predominant statistics used, unless otherwise stated.

## 5. Conclusions

Whereas cancer treatments continue to improve at a rapid pace, survival rates for PDAC patients have, unfortunately, not followed suit. In the case of PDAC, it has become increasingly clear that combinatorial strategies to eliminate tumor fibrosis may be requisite for therapeutic success. Increasing evidence also suggests that reducing levels of extracellular matrix (ECM) components not only relieves interstitial pressures to enhance drug delivery but may also modulate tumor-associated immune subsets to generate a tumor microenvironment more conducive to immunotherapy. Indeed, we and others have observed dramatic improvements in immunotherapy following direct depletion of hyaluronan and collagen. One major hurdle in targeting ECM components has been major adverse events associated with systemic, off-tumor toxicity. Incorporation of degradative enzymes into ST vectors represents a novel strategy to restrict ECM degradation to tumor tissue.

## Data Availability

The data presented in this study are available in “Collagenase-Expressing Salmonella Targets Major Collagens in Pancreatic Cancer Leading to Reductions in Immunosuppressive Subsets and Tumor Growth” and its associated supplementary materials.

## Supplementary Materials

The following are available online at https://www.mdpi.com/article/10.3390/cancers13143565/s1, Figure S1: SOT expression in attenuated ST, Figure S2: ST colonization and diffusion.
