# Supplementary material for: Collagenase-Expressing Salmonella Targets Major Collagens in Pancreatic Cancer Leading to Reductions in Immunosuppressive Subsets and Tumor Growth
Source: Cancers (Basel). 2021 Jul 16;13(14):3565. doi: 10.3390/cancers13143565 (PMC8306875; doi:10.3390/cancers13143565)
Supplement: Supplementary file 1 [file cancers-13-03565-s001.zip › cancers-1292862-supplementary.pdf]

cancers

MDPI

Supplementary Materials

Collagenase-Expressing *Salmonella* Targets Major Collagens in Pancreatic Cancer Leading to Reductions in Immunosuppressive Subsets and Tumor Growth

Nancy Danielle Ebelt, Vic Zamloot, Edith Zuniga, Kevin B. Passi, Lukas J. Sobocinski, Cari A. Young, Bruce R. Blazar and Edwin R. Manuel

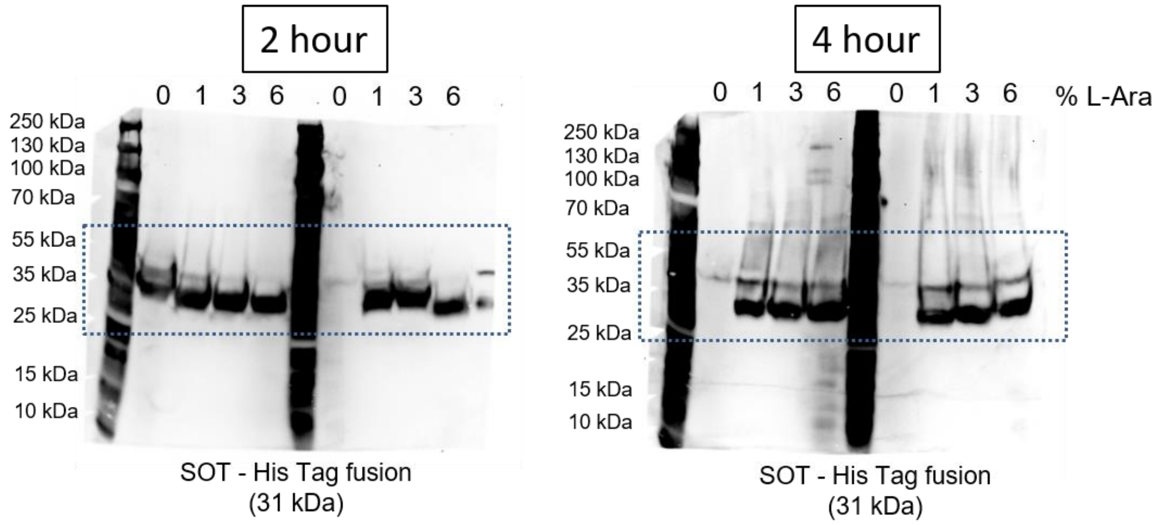

Figure S1. SOT expression in attenuated ST. ST-SOT was cultured in LB containing 0% (uninduced) or 1% to 6% (induced) L-arabinose for 2 or 4 hours at 37 °C. Lysates from pelleted bacteria were analyzed for SOT expression by western blot for the His-tag fused to the C-terminus of SOT (α-His).

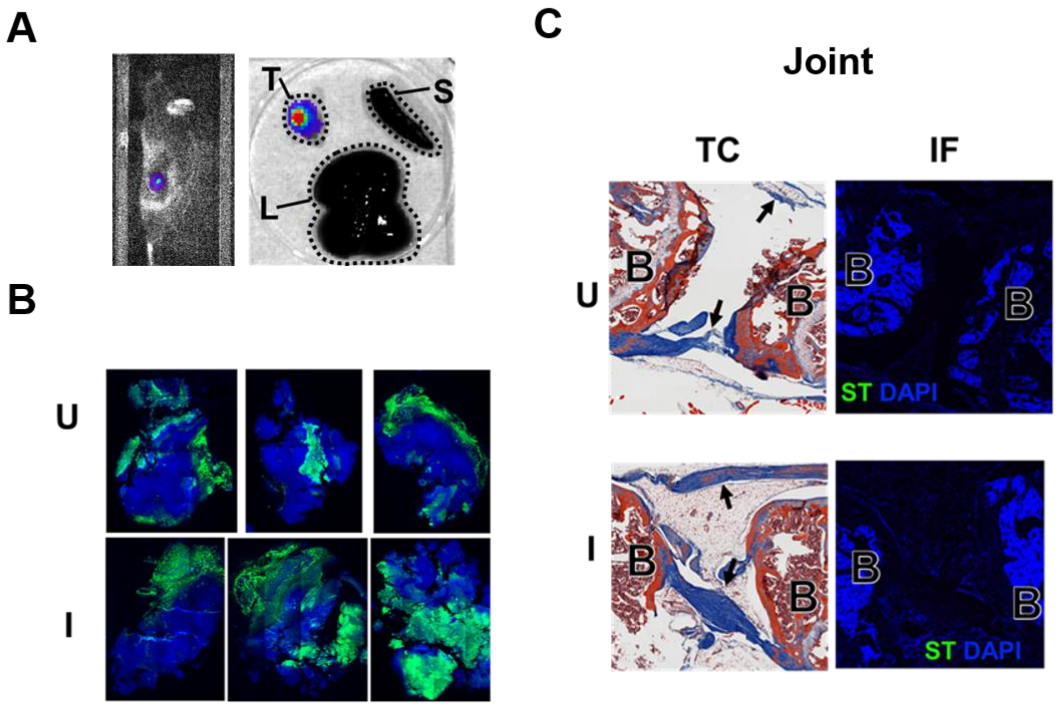

Figure S2. ST colonization and diffusion. **(A)** ST-LUX (5x106 CFUs) was i.v. administered into mice bearing o.t. KPC4662.5 tumors (>150mm^3). Forty-eight hours later, bioluminescence in mice were imaged by intravital (IVIS) imaging (left panel) and then euthanized to examine bioluminescence in tumor (T), spleen (S) and liver (L) (right panel). **(B)** ST-SOT diffusion (green) under uninduced (U) and induced (I) conditions was measured in Pan02 tumor sections by immunofluorescence. Tumor area approximated using DAPI staining (blue). **(C)** Pan02 tumor-bearing mice were treated with ST-SOT (U or I conditions) and 48 hours post-treatment, mice were euthanized and hind-leg joints were serial sectioned and stained by trichrome or by immunofluorescence to detect for presence of ST-SOT. DAPI, blue. B, bone.
